# In vitro anticancer effect of tricyclic antidepressant nortriptyline on multiple myeloma

**DOI:** 10.3906/biy-1802-11

**Published:** 2018-10-25

**Authors:** Ayşenur BİBER, İpek Z. DURUSU, Can ÖZEN

**Affiliations:** 1 Biotechnology Graduate Program, Central Laboratory, Center of Excellence for Biomaterials and Tissue Engineering, Middle East Technical University , Ankara , Turkey

**Keywords:** Tricyclic antidepressant, combination chemotherapy, cancer, apoptosis, multiple myeloma

## Abstract

Drug repurposing has been proved to be an effective strategy to meet the urgent need for novel anticancer agents for multiple myeloma (MM) treatment. In this work, we aimed to investigate the anticancer effect and mechanism of tricyclic antidepressant nortriptyline (NTP) on the U266 MM cell line. The in vitro inhibitory effect of NTP at various doses and time points was studied. The combination potential of cisplatin-NTP was also investigated. Cell cycle analysis and three flow cytometric apoptosis assays were performed. NTP showed dose- and time-dependent inhibitory effects on the U266 MM cell line. NTP had greater inhibitory effect than cisplatin (IC50 26 µM vs. 40 µM). The cisplatin-NTP combination is antagonistic. In addition to G2/M phase cell cycle arrest, NTP induced apoptosis as indicated by mitochondrial membrane potential and caspase-3 and annexin V assays. NTP has inhibitory and apoptotic effects on U266 MM cells. The cisplatin-NTP combination indicated strong antagonism, which may have significant clinical relevance since antidepressants are commonly employed in adjuvant therapy for cancer patients. Based on these findings, the therapeutic potential of NTP for MM treatment should be investigated with in-depth mechanistic studies and in vivo experiments.

## 1. Introduction

Multiple myeloma (MM) is a cancer of plasma cells. It is a complex hematological malignancy in which the interaction of neoplastic B cells with the bone marrow microenvironment plays a critical role in the progression of the disease. MM is the second most common blood cancer (10% of all blood cancers) and accounts for approximately 1% of all new cancer cases (Rajkumar, 2014) . Effective combination chemotherapy including proteasome inhibitors (bortezomib) and immunomodulatory agents (lenalidomide) has significantly increased the survival rate of patients (Mimura et al., 2015; Röllig et al., 2015) . However, a high rate of relapse, especially due to multidrug resistance, requires the addition of new drugs to existing chemotherapy strategies.

Tricyclic antidepressants (TCAs) belong to a broad class of psychoactive drugs. Interestingly, they also show potent in vitro and in vivo anticancer effects on a large variety of tumor cells such as colon, osteosarcoma, prostate, glioma, skin squamous carcinoma, and MM (Frick and Rapanelli, 2013) .

Nortriptyline (NTP), a classic TCA, also displays antineoplastic effects on osteosarcoma, prostate, melanoma, and bladder cancer (Hsu et al., 2004; Pan et al., 2010; Mao et al., 2011; Yuan et al., 2015) . Pan et al. showed that NTP inhibits proliferation and induces apoptosis in human prostate cancer (PC3) cells (Pan et al., 2010) . More recently, Yuan et al. investigated the therapeutic potential of NTP on bladder cancer and observed cell cycle arrest, intrinsic and extrinsic apoptosis, increase in reactive oxygen species production, and suppression of tumor growth in mice (Yuan et al., 2015) .

Based on previous findings on its antitumor potential and results from our screening studies, we investigated NTP’s potency and the mechanism of its anticancer effect on the U266 MM cell line.

## 2. Materials and methods

### 2.1. Chemicals

Clomipramine, imipramine, amitriptyline, maprotiline, and mianserin were purchased from Alfa Aesar (Lancashire, UK). Opipramol, protriptyline, nortriptyline, desipramine, and cisplatin were obtained from SigmaAldrich (Taufkirchen, Germany).

### 2.2. Cell vulture

Human MM cell line U266 was kindly provided by Professor Yusuf Baran (İzmir Institute of Technology, İzmir, Turkey) and maintained in RPMI 1640 growth medium supplemented with 10% fetal bovine serum, 1% penicillin/ streptomycin, and 2.5 µg/mL Plasmocin prophylactic at 37 °C in a 5% CO2 incubator. Drug treatment was performed at 105 cells/mL density.

### 2.3. Cell viability assays

Drug cytotoxicity screening, potency (IC50) determination, and time-response and combination assays were completed using the Promega (Madison, WI, USA) CellTiterBlue cell viability assay. For each sample, five technical replicates were prepared. Measurements were taken with a Molecular Devices (Sunnyvale, CA, USA) SpectraMax Paradigm fluorescence plate reader at 555 nm excitation and 595 nm emission settings. IC50 values were calculated with GraphPad Prism v5.0 (GraphPad Inc., La Jolla, CA, USA). Combination index (CI) values were calculated using CompuSyn software (ComboSyn Inc., Paramus, NJ, USA) (Chou, 2006) .

### 2.4. Flow cytometry

Cell cycle analysis and apoptosis assays were carried out with a BD Biosciences (San Diego, CA, USA) Accuri C6 flow cytometer.

### 2.5. Cell cycle analysis

Cells were treated with 15 µM NTP for 24 h and harvested. After a cold PBS wash, samples were fixed with 70% ethanol and kept for 2 h on ice. Following centrifugation and PBS washing, cells were stained with propidium iodide (25 µg/ mL) in PBS (30 min, 37 °C). The staining solution also contained 3 mg/mL RNAse. Samples were then analyzed with a flow cytometer.

### 2.6. Apoptosis assays

Cells were treated with 30 µM NTP and analyzed at time points of 12, 24, and 48 h with flow cytometry. Samples were prepared as described in the manufacturer`s procedures. To study the effect of NTP treatment on mitochondrial membrane polarization, the BD Biosciences MitoScreen Flow Cytometry Mitochondrial Membrane Potential Detection Kit was used. Active caspase-3 and phosphatidylserine detection was achieved with the PE Active Caspase-3 Apoptosis Kit and Annexin V Apoptosis Detection Kit I from the same manufacturer. Fluorescence micrographs were obtained using a Zeiss (Oberkochen, Germany) LSM 510 confocal laser scanning microscope equipped with PlanNeouflar 40x/1.3 Oil DIC objective.

### 2.7. Statistical analysis

For cytotoxicity screening and time-response and annexin-V assays, statistical significance of results was analyzed using GraphPad Prism with one-way ANOVA and the Tukey posttest module. Other experiments were evaluated using unpaired t-tests with two tails. Significances of differences are marked in figures with asterisks. Statistical analyses were conducted on data from three independent experiments.

## 3. Results

### 3.1. Potency screening of selected antidepressants

**Figure 1:**
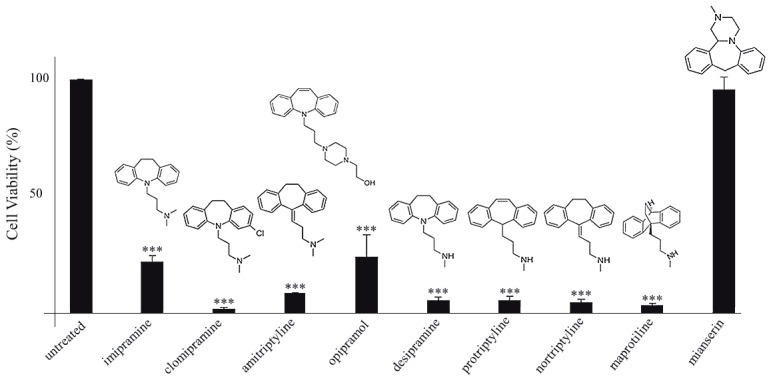
Effect of selected antidepressants (100 μM, 24 h) on viability of U266 cells. Drug structures are shown above the corresponding bars. Asterisks denote statistical significance at P < 0.0001 (n = 3).

We screened seven tricyclic (imipramine, clomipramine, amitriptyline, opipramol, desipramine, protriptyline, nortriptyline) and two tetracyclic (maprotiline, mianserin) antidepressants for their effects on the viability of the U266 MM cell line. Each drug was applied at 100 µM for 24 h. Except mianserin, all tested TCAs and maprotiline showed significant inhibitory effects on cell growth (Figure 1). Imipramine and opipramol exhibited relatively weaker inhibition compared to others. The tricyclic side chain seems to have a critical role since its drastic modification (opipramol) or removal (mianserin) diminished the effect on cell viability. Among the positive hits, we selected NTP for further in vitro characterization. To our knowledge, this is the first report investigating the mechanism of NTP’s effect on MM.

### 3.2. Nortriptyline shows dose- and time-dependent toxicity on U266 cells

**Figure 2:**
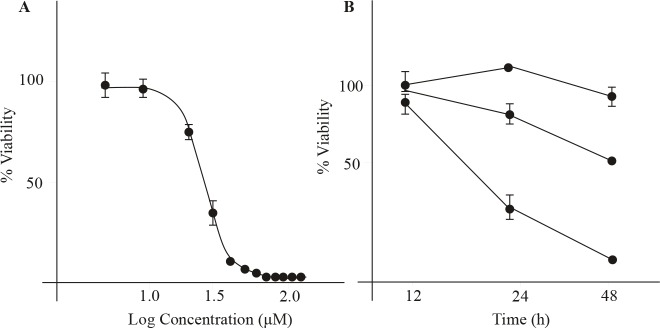
Dose and time response of NTP’s inhibitory effect on U266 cell viability. A) Dose response and potency (IC50) determination (n = 3). Concentration range was 1.0 to 120 μM. B) Time response (12, 24, and 48 h) of nortriptyline treatment at 5, 15, and 30 μM. Asterisks denote statistical significance at P < 0.001 (n = 3).

Dose and time dependences of NTP’s effect on U266 cells were examined in the range of 1 µM to 120 µM and at time points of 12, 24, and 48 h. We also calculated the IC50 of NTP at 24 h (Figure 2A). The potency of cisplatin (cis), an anticancer drug currently in clinical use for MM treatment, was also determined for comparison. The IC 50 values of NTP and cis were determined as 26.1 ± 1.0 and 39.8 ± 9.9. The solubility problem of cis made it quite difficult to reduce the variation among the biological replicates, resulting in a higher error in calculation. Keeping this in mind, NTP seems to be a more potent agent against MM. As expected, longer NTP treatment corresponds to higher inhibitory effects on cell viability (Figure 2B).

### 3.3. Nortriptyline-cisplatin combination is antagonistic

Cancer treatment efficiency significantly increases when drugs are used in combination. Based on the promising anticancer potential of NTP shown in the previous section, we decided to test the cis-NTP combination on MM cell viability. The molar ratio of the mixed drugs is a critical factor determining the type (synergistic, antagonist, or additive) and strength of the combination effect (Tsakalozou et al., 2012) . In this study, we used a simple approach with only four combinations as listed in the Table. Results were analyzed with CompuSyn software. Interestingly, all four cis-NTP combinations resulted in strong antagonism as indicated by the corresponding CI values.

**Table:** Inhibitory effect of NTP, cis, and cis-NTP combination at 24 h (n = 2). Combination index (CI) < 1 (synergy), CI = 1 (additivity), CI > 1 (antagonism).

NTP (μM)	Cis (μM)	Combination cytotoxicity (%)	Combination index (CI)
6	6	8.1 ± 0.3	1.8 ± 0.2
15	15	26.9 ± 1.6	2.0 ± 0.0
30	30	65.5 ± 1.7	1.7 ± 0.3
45	45	84.7 ± 3.0	1.5 ± 0.2

### 3.4. Nortriptyline arrests U266 cell cycle at G2/M phase

**Figure 3:**
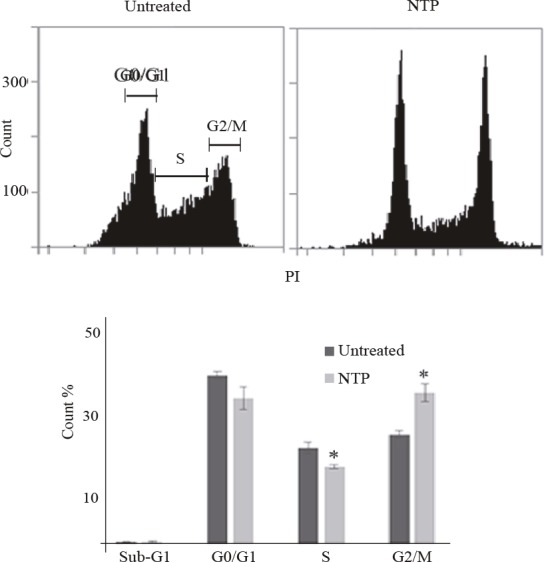
Effect of nortriptyline treatment (15 μM, 24 h) on U266 cell cycle. A) Representative flow cytometry fluorescence intensity histograms of cells stained with propidium iodide. Intensity ranges for corresponding cell-cycle phases (G0/G1, S, and G2/M) are labeled. B) Bar plots of normalized count values of each phase for untreated and nortriptyline-treated cells (n = 3). Error bars (1%–3%) indicate standard error of mean. Asterisks denote statistical significance at P < 0.05.

The inhibitory effect of NTP on U266 cells may arise from antiproliferative and/or cytotoxic mechanisms. We first investigated antiproliferative effect by analyzing the progression of cell cycle with propidium iodide (PI) staining and flow cytometry after NTP treatment (15 µM, 24 h). Distribution of untreated and NTP-treated cells at G1 (44.9% vs. 38.7%), S (25.5% vs. 20.5%), and G2/M (29.1% vs. 40.2%) phases of cell cycle are provided in Figure 3. According to these results, there is a statistically significant difference in the G2/M populations of untreated and NTPtreated cells, indicating a drug-induced cell cycle arrest.

Based on previous TCA studies, we expected NTP to induce apoptosis. In the following part, three common apoptotic biomarkers were probed to test this strong possibility.

### 3.5. Nortriptyline causes mitochondrial membrane depolarization

**Figure 4:**
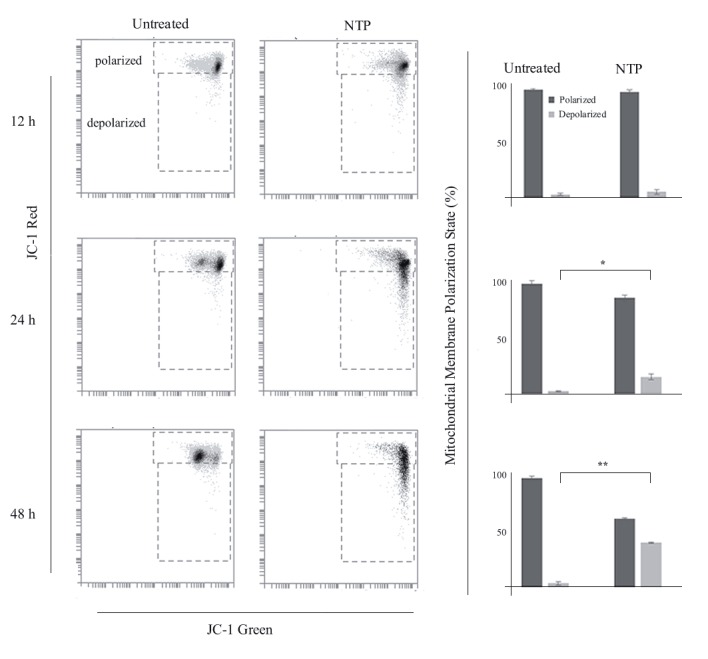
Effect of nortriptyline (30 μM) on mitochondrial membrane potential as a function of treatment time (12, 24, and 48 h). A) Representative flow cytometry fluorescence intensity dot plots of cells stained with JC-1. Gated fluorescence intensity values for polarized and depolarized states are labeled. B) Bar plots of normalized mitochondrial membrane polarization state values for untreated and nortriptyline-treated cells (n = 3). Error bars (1%–3%) indicate standard error of mean. Asterisks * and ** denote statistical significance at P < 0.05 and P < 0.01, respectively

Mitochondria play a critical role in programmed cell death and depolarization of the mitochondrial membrane was shown to be one of the early events of apoptosis in some earlier cases (Salvioli et al., 1997) . To investigate the effect of NTP (30 µM) on mitochondrial membrane potential, we used JC-1 staining and analyzed the treated cells at 12, 24, and 48 h with flow cytometry. Red-to-green shift of the JC-1 (5,5’,6,6’-tetrachloro-1,1’,3,3’-tetraethylbenzimidazol carbocyanine iodide) fluorescence in the flow cytometry histograms (Figure 4) was used as an indicator of the mitochondrial health.

The majority of the control and NTP-treated cell populations had healthy mitochondria (polarized membrane) at 12 h. However, the depolarization signal of the drug-treated cells started to increase at 24 h (3% control vs. 15% NTP) and became almost tenfold higher at 48 h (4% control vs. 39% NTP).

### 3.6. Nortriptyline increases caspase-3 activity

**Figure 5:**
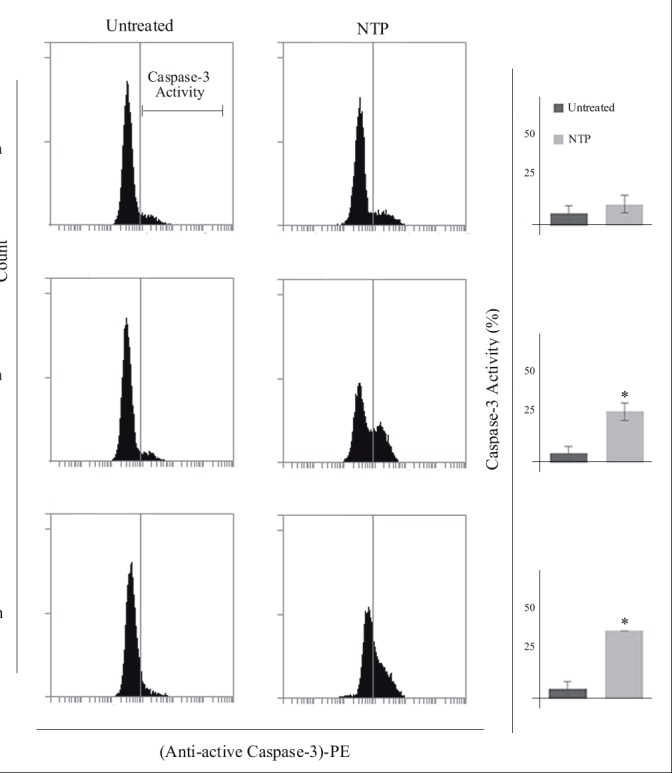
Caspase-3 activity of nortriptyline-treated (30 μM) U266 cells at 12, 24, and 48 h. A) Representative flow cytometry fluorescence intensity histograms of cells stained with antiactive caspase-3 PE. Intensity threshold for caspase-3 activity is indicated in the upperleft panel. B) Bar plots of corresponding histograms (n = 3). Error bars (4%–5 %) indicate standard error of mean. Asterisks denote statistical significance at P < 0.05.

Caspase-3 is a key protease activated in both intrinsic and extrinsic apoptotic pathways at an early stage. We used an immunoflourescence-based caspase-3 assay to specifically detect the active form of the protease as a second apoptotic biomarker. NTP treatment had no significant effect on active caspase-3 level at 12 h (Figure 5). However, druginduced significant rises were observed at 24 h (5% control vs. 28% NTP) and 48 h (6% control vs. 35% NTP).

### 3.7. Annexin-V assay also indicates nortriptylineinduced apoptosis

**Figure 6:**
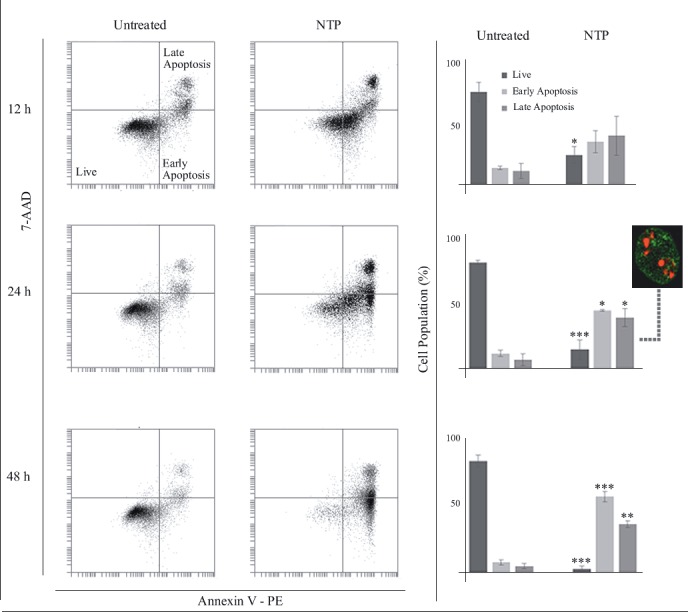
Flow cytometry analysis (12, 24, and 48 h) of annexin V-PE/7-AAD-stained U266 cells treated with 30 μM nortriptyline. A) Representative dot plots of annexin V-PE vs. 7-AAD signals gated as live, early apoptotic, and late apoptotic quadrants. B) Cell population bar graphs of corresponding dot plot quadrants (n = 3). Fluorescence micrograph of a late apoptotic cell is also shown for 24 h NTP treatment. Asterisks *, **, and *** denote statistical significance between control and treatment populations at P < 0.05, P < 0.01, and P < 0.001, respectively

As the last apoptotic indicator, we employed annexin-V, which is based on the loss of cell membrane phospholipid asymmetry. As labeled in the dot plot (Figure 6), healthy cells are PE-annexin V-negative and 7-AAD-negative, early apoptotic cells are PE-annexin V-positive and 7-AAD-negative, and late apoptotic/dead cells are PEannexin V-positive and 7-AAD-positive. Starting at 12 h, NTP treatment caused a significant decrease in the healthy cell population (76% control vs. 24% treatment). As the treatment time extended to 24 and 48 h, increases in the percentage of early and late apoptotic cells (~4-fold and 6-fold in 24 h, ~7-fold and ~8-fold in 48 h, respectively) also became significant. Fluorescence microscopy observations (Figure 6B, boxed micrograph) also confirmed flow cytometry findings. Collectively, all three assay results show that NTP induces apoptosis in U266 cells.

## 4. Discussion

Results on the inhibitory effect and mechanism of NTP reported in this work agree with previous studies that strongly supported the anticancer effect of antidepressants (Frick and Rapanelli, 2013) .

NTP has dose- and time-dependent inhibitory effects on U266 MM cells. Similar results were obtained by others on osteosarcoma, prostate, melanoma, and bladder cancer cells (Hsu et al., 2004; Pan et al., 2010; Mao et al., 2011; Yuan et al., 2015) . IC50 values of NTP on MM cells (26 µM) and bladder cancer cells 40 µM, (Yuan et al., 2015) are similar. Within 24 h, NTP arrested the cell cycle, caused mitochondrial membrane depolarization, increased active caspase-3 levels, and induced loss of cell membrane asymmetry. These observations confirm both antiproliferative and apoptotic effects of the drug on U266 cells.

Yuan et al. found that NTP has less than 20% inhibitory effect on peripheral blood mononuclear cells at a high concentration (100 µM). In addition, Mao et al. reported that amitriptyline, the methylated form of NTP (Figure 1), was not cytotoxic to normal blood cells (Mao et al., 2011) . These findings and the fact that NTP is an approved drug support the safety of its potential use in cancer therapy. Comparison of in vitro potency and in vivo serum concentration is another important point that needs addressing. Approximately 10 µM NTP was detected in the serum after daily administration at typical doses up to 75 mg (Yeragani et al., 2012) . This concentration is about onethird of the calculated IC50 value. Therefore, at this point, it is quite difficult to judge the clinical potential of NTP for MM treatment without additional in vivo experiments.

NTP in combination with cis was shown to be antagonistic within the tested dose regime. This is a clinically relevant finding since adjuvant therapy with antidepressants is commonly used for cancer patients (Kabolizadeh et al., 2012) . In recent years, some other antidepressants (paroxetine, fluoxetine, and bupropion) were also shown to have a negative effect on a commonly used cancer drug, tamoxifen, in breast cancer patients by inhibiting the metabolic conversion of the drug into its active form (Desmarais and Looper, 2009; Kelly et al., 2010) . Based on our results and the tamoxifen example, we suggest that NTP may also have a negative impact on the clinical effectiveness of cis-containing chemotherapy regimens. On the other hand, there are also examples revealing the synergistic effect of TCAs in combination with other drugs. Amitriptyline combinations with dexamethasone and bortezomib were shown to have synergy on myeloma and MM cells (Mao et al., 2011; Zhang et al., 2013) . Similarly, combining clomipramine with LiCl or imatinib also resulted in a synergism on neuroblastoma and glioma cells (Bilir et al., 2008, 2010; Zhang et al., 2013) .

There are at least two reported cases in which cis combinations with getfiinib and fingolimod were antagonistic (Tsai et al., 2011; Zhang et al., 2013) . To our knowledge, the cis-NTP combination was not previously investigated; however, we found a study by Kabolizadeh et al., which involves desipramine, a structural analog of NTP (Figure 1) (Kabolizadeh et al., 2012) . In this work, desipramine (5 to 50 µM) highly enhanced the cytotoxicity of cis (1 to 15 µM) on colorectal carcinoma cell lines with CI values reported in from 0.174 to 0.922.

The antagonistic effect of the cis-NTP combination may have more than one explanation. Direct drug– drug interaction may be one of these, although such an interaction was not evident in the cis-desipramine case as shown by nuclear magnetic resonance experiments (Kabolizadeh et al., 2012) . NTP might have caused an increase in the DNA repair mechanism, which would diminish the susceptibility of cis-induced DNA damage. NTP may also reduce the cellular accumulation of cis, as in the case of cis-Raf kinase inhibitor BAY 43-9006 combination (Heim et al., 2005) . In this scenario, most likely the NTP target would be organic cation transport machinery, which was previously proposed for cellular cis uptake (Koepsell et al., 2007; Hall et al., 2008) . Another possibility is the activation of conflicting signaling pathways by cis and NTP. A related TCA, imipramine, has been previously shown to induce autophagy in glioma cells (Jeon et al., 2011) . Induction of autophagic machinery by NTP might have interfered with the apoptotic pathway of cis-induced cell death.

NTP arrested U266 cells at the G2/M phase of the cell cycle. Yuan et al. also reported cell cycle arrest at G0/ G1 and G2/M phases in NTP-treated human and mouse bladder cancer cells (Yuan et al., 2015) . Amitriptyline (20 µM, 24 h) arrests KMS11 and LP1 MM cells at G0/ G1 by downregulating cyclin D expression and increasing expression of cyclin-dependent kinase inhibitors p27 and p21 (Mao et al., 2011) . Overexpression of p21 and p27 and cell cycle arrest was also reported in skin squamous carcinoma Ca3/7 cells treated with desipramine (Kinjo et al., 2009) . Finally, fluoxetine-induced G0/G1 arrest in lung and colon tumor cells was shown to involve cyclin A, cyclin D1, p21, and p53 (Stepulak et al., 2008) . As summarized above, all TCAs seem to have cell cycle arrest effects on cancer cells. Is there a significance of the phase in which the cells are arrested? Ruetz et al. and DiPaola both reached the conclusion that G2/M arrest is less well tolerated by the cells, leading to increased apoptotic outcome (DiPaola, 2002; Ruetz et al., 2003) . This evaluation provides a therapeutic advantage for NTP, which causes G2/M arrest in U266 cells.

We also showed that NTP induces apoptosis in MM cells. Various other TCAs were all reported to have the same effect on tumor cell lines (Frick and Rapanelli, 2013) . In particular, NTP induced caspase-dependent apoptosis in bladder cancer cells by both mitochondria and death receptor-mediated pathways (Yuan et al., 2015) . Zhang et al. studied amitriptyline triggered apoptosis in MM xenograft models (Zhang et al., 2013) .

In this work, we showed dose- and time-dependent inhibitory effects of the antidepressant drug nortriptyline on the U266 MM cell line. It was also demonstrated that in vitro potency of NTP was greater than that of cisplatin, a well-known cancer drug. Previous studies on NTP showed minimal or no toxicity to normal blood cells and in vivo antitumor effects. Our study provides the first in vitro evidence of NTP-induced cell cycle arrest and apoptosis in U266 cells. The molecular mechanism of both outcomes should be elucidated and compared to those of other antidepressants. Also, based on the reported results, NTP warrants further investigation using in vivo MM models. Antidepressants are commonly used in adjuvant therapy for depression and pain relief. Therefore, antagonism of the cis-NTP combination was another clinically significant finding in this work. In future studies, additional dose combinations can be tested to investigate the changes in the type and strength of the combination effect.

## Acknowledgment

Financial support for this work was provided by Graduate School of Natural and Applied Sciences, Middle East Technical University.
